# Supplementary material for: Are carbon emissions trading and green financial instruments synergistic? -Comprehensive quantitative research based on content analysis
Source: PLoS One. 2024 Mar 7;19(3):e0298601. doi: 10.1371/journal.pone.0298601 (PMC10919601; doi:10.1371/journal.pone.0298601)
Supplement: S1 Data — (ZIP) [file pone.0298601.s001.zip › Data/地级市Z_F工作报告.zip!/╣π╢1⁄2/╔ε█┌╩╨/└┤╫╘╣1⁄2╓┌║┼í╛┬φ┐╦╩2╛▌═°í┐_2008.docx]

各位代表：

　　现在，我代表深圳市人民政府向大会作政府工作报告，请予审议，并请各位政协委员和其他列席人员提出意见。

　　一、2007年工作回顾

　　2007年，我们以邓小平理论和"三个代表"重要思想为指导，以科学发展观统领全局，加快转变经济发展方式，努力构建社会主义和谐社会，圆满完成了市四届人大三次会议确定的目标任务，全年实现本市生产总值6765.4亿元，增长14.7%；人均GDP在内地大城市中率先突破1万美元，达到10628美元。在中国社科院等权威机构公布的科学发展、和谐发展、城市综合竞争力等评估报告中，深圳均名列内地城市榜首。

　　（一）效益深圳建设迈上新台阶。全市每平方公里土地产出GDP达3.46亿元，比上年提高0.48亿元。地方财政一般预算收入一年净增157亿元，达658亿元，增长31.4%；对中央财政的贡献大幅增长78.9%，上划中央收入突破2000亿元，达2112亿元。工业经济效益综合指数189.8%，提高3个百分点；工业企业实现利润总额增长10.4%。万元GDP能耗在不到全国平均水平一半的基础上继续有所下降；万元GDP水耗27.7立方米，下降7%，相当于世界平均水平的一半；全市二氧化硫和化学需氧量排放量分别下降6.9%和4.7%，空气质量优良天数比例达98.9%。循环经济推广月活动和建筑节能示范试点全面展开，我市被确定为国家发展循环经济示范城市和国家机关办公建筑及大型公共建筑节能检测示范城市之一。

　　产业结构不断优化。工业保持平稳增长，第三产业比重上升，三次产业结构比例为0.1∶50.9∶49.0。全年实现工业增加值3270.1亿元，增长15.1%。高新技术产品产值7598.8亿元，增长20.5%，其中具有自主知识产权的产品占58.6%。产业高端化取得突破性进展，中芯国际在深圳设立南方总部并投资建设8英寸和12英寸集成电路芯片生产线项目，深超光电第5代TFT-LCD面板生产线厂房封顶，深圳IT产业正式告别缺“芯”少“魂”的历史，跻身国内集成电路和新型平板显示生产发展的前沿地带。国内最大的外商投资生物制药项目赛诺菲-巴斯德流感疫苗项目正式签约。世纪晶源化合物半导体、意法半导体封装测试等一批高端项目推进顺利。产业适度重型化成效显著，比亚迪汽车研发中心建成启用，F6汽车正式下线，哈飞、标致合资项目积极推进，汽车产业集群及相关产业链初步形成。优势传统产业集聚基地建设加快。

　　金融业步入新一轮加速发展轨道。全市金融企业实现税前利润759亿元，增长超过2倍，金融机构存贷款余额双双突破万亿元，四大金融产业基地开工建设，32家中小企业成功在境内外上市，我市在深交所中小企业板挂牌的上市公司已达22家，占全国的10%。成功发行全国首只中小企业集合债券，民营中小企业融资环境进一步优化。平安保险成为我市首家跻身世界500强的本土企业，在我国世界500强非国有企业中排在首位。

　　高端服务业和总部经济加快发展。完善了高端服务业和总部经济发展的政策环境。物流业创下两个2000万的佳绩，深圳港集装箱吞吐量达到2110万标箱，机场旅客吞吐量2062万人次。制定文化产业发展规划纲要和“十一五”规划，文化产业园区建设稳步推进，文化软实力增强。国内资本金最大的新兴寿险公司生命人寿公司总部迁入我市，阳光保险集团公司注册成立，南方电网保险公司、国际公司落户深圳。商贸、旅游、会展等服务业健康发展。社会消费品零售总额1915亿元，增长14.6%；接待过夜入境游客 831.3万人次，增长16.6%。全年共举办各类展会72个，平均每5天一个展会，初步形成了与四大支柱产业相配套的展会体系。

　　（二）国家创新型城市建设取得新进展。我市与科技部、信息产业部、国家知识产权局和省政府分别签署协议，共建国家创新型城市、国家信息产业基地自主创新示范城市和知识产权强市，并被国家有关部委确定为综合性国家高新技术产业基地和国家知识产权示范城市创建市。全社会研发投入占GDP的比重达到3.34%。在国内率先出台地方知识产权指标体系，知识产权保护力度加大。全市专利申请量达到35808件，增长20.5%，其中发明专利19198件，增长31.7%，连续两年居全国首位。PCT国际专利申请2170件，增长30.6%，占全国总量的39.8%，连续四年居全国首位。新增“中国名牌产品”22个，“中国世界名牌产品”1个，“中国驰名商标”4件，广东省著名商标32件，均居全国、全省前列。其中我市拥有的中国世界名牌数量占全国的三成，居内地城市第一位。华为、中兴、迈瑞公司的三项技术成果获得国家科技进步二等奖，深圳华大基因研究院成功绘制第一个完整的中国人基因组图谱“炎黄一号”。中科院深圳先进技术研究院7大研究平台正式启动。与香港签订“深港创新圈合作协议”，共同建设一个跨城市、一体化的区域创新体系和产业聚集带，去年深港两地共同投入资金5500万元，支持创新项目53个。成功举办2007中国国际人才交流大会，搭建起引进国际化人才和创新型人才的新桥梁。

　　（三）城市现代化建设呈现新面貌。完成全社会固定资产投资1345亿元，其中基本建设投资714.1亿元，增长11.6%。一批事关城市长远发展和提升辐射力、竞争力的重大基础设施建设取得较大进展。广深港客运专线、厦深铁路正式动工；城市轨道交通建设掀起高潮，轨道交通二期工程5条线路全面开工；深港西部通道、福龙路、南光高速公路建成投入使用，盐坝高速公路C段、深盐二通道等工程加快推进，南坪快速路二期、丹平快速路一期、沿江高速、205国道改造等项目动工建设；大铲湾港区正式开港，一个现代化新港区正在形成。全市新建成5个集装箱泊位并投产使用，铜鼓航道一期工程完工，深圳港的国际集装箱枢纽港地位进一步巩固。机场飞行区扩建工程进展顺利。

　　资源能源保障体系建设成效显著。东江水源二期、北线引水工程、铁岗水库扩建、公明供水调蓄工程等一批水源、水库工程建设加快，大鹏、梅林等支线工程基本完工。有效整合特区外水资源，加强全市水源的统一管理和科学调配，全年供水量超过18亿立方米，在枯水之年保证了生产生活正常用水。全年投入40多亿元加强电网建设，贵广二回直流配套工程等一批输变电关键项目完工投产。与南方电网建立战略合作关系，积极争取省网用电支持，鼓励地方机组发电，实施错峰用电和节能节电措施，在电网最高负荷突破1000万千瓦的情况下基本保证了城市电力供应，成为全省供电形势最好的城市之一。加强与成品油供应商的协调合作，成品油市场供应基本正常。广东大鹏LNG一期增量工程竣工，深圳LNG项目完成选址，争取西气东输二线向我市供应天然气工作取得重大进展，城市天然气利用、天然气转换等工作加快推进。粮油等重要物资储备体系逐步健全，城市运行的安全保障能力大大增强。

　　城市规划管理水平不断提高。高起点规划建设光明新城、龙华新城、大运新城和坪山新城，拉开城市发展布局，特区内外一体化建设呈现新局面。描绘未来十年建设蓝图的城市总体规划和土地利用总体规划修编工作取得重大进展。各层次规划编制工作有序推进，地下空间利用规划取得积极进展。与香港联合成功举办第二届深港城市/建筑双城双年展。“基层基础年”和“城市管理年”活动走向深入，城中村和社区环境综合整治加快推进，顺利通过国家卫生城市复查，违法建筑查处工作继续保持了高压态势，户外广告整治成效明显，“重民生，保水缸”专项行动强力推进，闲置土地清理工作有效开展，覆盖全市的数字化城管系统全面建成。高质量完成一批重点道路改造工程，深南大道更加安全畅通、更加赏心悦目，成为城市的靓丽风景线。适应城市管理转型要求，市政府代表团和10个专项小组大规模赴新加坡深度学习考察先进城市管理经验，考察成果转化成为加强生态文明建设的3年行动纲领以及9个行动方案、80个具体工作项目，计划通过三年时间的持续推进，大大提升城市的品位和内涵。

　　生态市建设全面启动。认真组织实施《关于加强环境保护建设生态市的决定》，成为首个“国家生态园林示范城市”。“蓝天行动”成效显著，在全国首批实现了燃煤机组全部脱硫，电厂“油改气”工作加快推进，南头半岛、清水河等重点片区空气污染治理工程初见成效，南头半岛二氧化硫年日均浓度下降18%。国Ⅲ排放标准全面实施，机动车尾气污染减轻。治污保洁工程强力推进，污水处理厂及配套管网建设加快，新建污水主干管网140公里。持续推进特区内河道生态景观建设和跨市河流水环境综合整治，"五河一湖"治理工作取得实质性进展。饮用水源水质达标率99.4%。

　　（四）改革开放迈出新步伐。经济体制改革进一步深化。市属国有企业三项制度改革、转换内部经营机制实现新突破，做大做强一批企业集团工作取得重要进展，国有独资公司董事会建设试点进展顺利，市属国有企业利润总额超过200亿元，再创历史新高，各项经济效益、质量指标均位居内地大城市前列。实施中小企业上市培育工程，111家民营领军骨干企业获得认定和重点扶持，民营及中小企业发展环境改善。

　　社会管理体制改革加快。事业单位分类改革工作全面完成，转企的事业单位和划转的党政机关所办企业已纳入规范的国有资产监管体系中，结束了政府机关和事业单位办企业的历史。事业单位内部机制改革、法定机构试点有序推进，公共服务开始朝着标准化、均衡化、普惠化方向发展。居住证制度试点工作顺利完成，盐田区逾19万人成功申请了集劳动社保、计划生育、教育、公共交通等行政管理和公共服务功能于一卡的居住证。进一步完善了社会组织管理，推进公民社会建设。实施社工工作试点，更好地满足个性化的社会服务需求。

　　行政管理体制改革力度加大。以创新的体制设立光明新区，探索城市化与工业化良性互动、促进区域协调发展的新模式。完成非行政许可清理，非行政许可事项减少到348项，减幅达50%。出台《深圳市政府绩效评估指标体系》，在6区和16个政府直属单位开展绩效评估试点。建立政府部门责任检讨及失职道歉制度，加大行政问责力度，203人和37个单位被追究行政过错责任。完成全市行政事业单位资产清查工作，全面整合各类政府性专项资金，清理预算单位自有账户结余资金，财政性资金统筹调度、统一管理得到加强。公安专业化改革有序推进，一线执法力量加强，警队的整体战斗力提高。城市街道综合执法体制改革在试点基础上全面推开，通过整合行政资源，完善执法机制，初步解决了多头执法、权责分割、执法效率不高、基层执法薄弱等问题。开展聘任制公务员制度试点，公开招聘首批聘任制公务员。

　　政府行政执行力明显提高。强化政府目标管理，扩大了部门责任“白皮书”制度实施范围，根据去年政府工作报告分解的169项重点工作较好完成。成立深圳市行政电子监察评价中心，充分发挥市行政服务大厅在机制创新和资源整合方面的优势，建立统一受理、协同办理的工作机制，提高了审批效率，行政审批效能测评名列全省第一。市政府会议、发文减少15%的目标基本实现。

　　民主法制建设进一步加强。坚决执行人大及其常委会决议，在人大的监督和支持下开展工作。加强与人民政协联系，完善与人民群众联系渠道，提高了决策民主化、科学化水平。办理市人大重点建议2件，人大代表建议533件，政协提案605件，代表和委员满意和基本满意率达到99%以上。坚持依法治市，切实推进政府法制建设，制定和修订政府规章15项，提请人大通过和修订地方法规8项。

　　大力实施“走出去”战略。建立企业“走出去”政策支持和配套服务体系，越南中国（深圳）经贸合作区项目扎实推进，深圳企业境外协议投资额增长32%，对外工程和劳务合作营业额增长54.8%。外贸出口结构和引进外资结构得到优化，外贸进出口总额2875.3亿美元，增长21.1%，其中外贸出口总额1684.9亿美元，增长23.8%，实现出口总额十五连冠。全市WTO事务工作体系基本形成，进出口产业损害预警系统投入运行。首次编制《深圳市对口合作与服务全国"十一五"规划》，与湖南、江西签订经济社会合作框架协议，建立了一批异地深圳工业园和产业转移园，与中西部地区、东北地区、华南地区在基础设施、能源、劳务等方面的合作全面加强。

　　深港、深澳合作进入历史最好时期之一。按照中央要求圆满完成庆祝香港回归十周年各项工作，胡锦涛主席亲自为西部通道开通剪彩。深圳湾口岸和福田口岸正式开通。与香港特别行政区政府签署了“1＋6”合作协议，形成了更紧密的合作关系，与澳门建立双方高层沟通协商机制，深港、深澳全方位合作深度推进。

　　（五）和谐深圳建设取得新成果。全市公共服务和民生领域财政一般预算支出达到528.3亿元，占一般预算总支出的72.6%。政府投资项目中民生领域总计投入200.6亿元，占政府投资总规模的71.8%。全市人均可支配收入达到24870元，继续居内地大城市首位。积极打造充分就业城市，建立了“零就业家庭”灭零工作长效机制，全年帮助3万多名失业人员实现就业再就业，全市登记失业率保持在2.29%的较低水平。政府对每个少儿每年补贴75元建立少儿医保制度，从而在全国率先初步实现“全民医保”。实施《深圳市社会医疗保险办法》，扩大保障范围，提高保障水平，劳务工医保住院起付线降低100元，劳务工医疗保险门诊药品目录由1200种增加到1841种。劳务工医疗保险参保人数达到595万，工伤保险参保人数达到675万，均居全国首位。出台加强住房保障的系列政策，在全市范围内强力开展政策性出租住房清退工作，共清退政策性出租住房1899套。住房保障工作制度化、规范化水平进一步提高。加强房地产市场管理，房地产价格过快增长的势头得到有效遏制。打击恶意欠薪取得成效，农民工就业管理服务加强，被评为“中国最受农民工欢迎的十大城市”之一。最低工资标准和低保标准进一步提高。居民消费价格总水平上升4.1%，低于全国平均水平。

　　全市教育经费投入128.8亿元，其中财政性教育经费投入93.8亿元。新增中小学学位3.27万个，并逐步建立起学校设施和生均经费标准化配置制度，全市210所学校开展互助共建，教育均衡发展取得成效。非户籍人口子女就读管理进一步规范，全市义务教育新生17.2万人中，非户籍人口子女占68%。民办教育规范发展。高等教育、职业教育发展加快，南方科技大学正式启动建设，市属高校改扩建工程和教学管理体制改革进一步推进，大学城科研和人才培养能力提升。公共卫生重大项目建设进展顺利，社区健康服务制度进一步完善。财政补贴3.9亿元规范调整医疗服务价格，医疗服务总体价格水平下降8.1%。突发公共卫生事件应急处置能力增强，公共卫生服务水平和质量稳步提高。公共文化服务体系不断完善，举办一系列高水平的文艺演出和品牌文化节庆活动，市属公益性文化场馆免费向社会开放，深圳音乐厅落成开业，文艺精品创作喜获丰收，《亲情树》等4部作品获得全国“五个一工程”奖。成功申办第26届世界大学生运动会，各项筹备工作和场馆建设全面推进，大运文化节拉开帷幕，顺利举办了一批国际高端赛事。

　　根据市民和人大代表的建议，政府制定实施了公交降价与公交补贴方案，财政每年补贴6亿元左右，使市民刷卡乘车的费用平均下降25%以上。对实施初期出现的一些问题，政府高度重视，积极协调，并加快特区外公交企业重组和资源整合。目前公交降价的各项措施已基本落实，降价幅度和市民优惠数量已达到预期目标。

　　社会治安形势进一步好转，全市刑事案件下降5.5%。全面排查化解矛盾纠纷，及时处理涉及民生的信访突出问题，积极推动“大调解”体系建设，加强城市应急、“三防”等工作，有力地维护了社会稳定。稳步推进食品安全“五大工程、四大体系”建设，加强菜篮子食品生产、加工、流通管理，对生猪生产和基地回流农产品实施补贴，稳定了生猪等副食品的市场供应，较好地保证了市民生活需要。财政还拿出1000多万元对低保户和市属大中专院校学生进行补贴。全市最大的菜篮子工程深圳国际农产品物流园动工建设。大力开展产品质量和食品安全专项整治，顺利通过国家检查，全市食品中毒事件下降36.8%。加强市场监管，深入开展"清无"行动，专项打击黑网吧、非法营运、套牌车等违法行为，平稳完成2000辆出租车投放工作，平衡了市场供求关系，市民“打的难”问题有所缓解。市财政安排1000万元启动我市道路交通事故社会救助基金。加强安全生产工作，安全生产事故下降20.1%，死亡人数下降5.1%。在"全国宜居城市"和城市生活质量评比中，深圳均名列第一。

　　社区管理体制改革进展顺利，社区信息化工作全面推进。人口和计生工作加强，流动人口政策生育率提高9.57个百分点。社会福利、慈善和残疾人事业加快发展，外事、侨务、港澳台务、民族宗教、统计、档案、民防、气象等各项事业都取得新进步，连续第四次获得"全国双拥模范城"光荣称号。

　　（六）十件民生实事全面完成。去年《政府工作报告》确定的民生实事全部按时完成，向全市人民交出了一份合格的答卷。新建124家社康中心、343座垃圾转运站，建成104个社区公园；全市劳动合同签订率达到92.5%；新增政策性住房用地60.5万平方米，开工、在建2.82万套经济适用房和公共租赁房，可供租售量达6006套；少儿医疗保险参保人数达到33.5万；特区外96所原村办小学设备设施配置全面完成，财政补助经费1.7亿元，校舍改扩建工程全面启动；两家豆制品厂主体工程完工、三家大型屠宰场开工建设、农贸市场升级改造率达到50%；特区内建成区基本实现500米公交全覆盖，特区外建成区500米公交覆盖率达到65.7%，福田交通综合枢纽换乘中心建成投入使用；12345政府公开电话系统正式启动。

　　各位代表，今年春节前后，我国南方一些地区遭遇历史罕见的低温雨雪冰冻灾害。面对这场重大考验，在党中央国务院、省委省政府和市委的坚强领导下，我们紧急动员、周密安排，安全妥善地完成了春运工作任务，全力支持和配合湖南、湖北、贵州等灾区的抗灾工作，鼓励和帮助数百万来深建设者留在深圳过年。在全市上下的共同努力下，春节期间全市居民生产生活秩序井然，社会保持和谐稳定，让全市人民度过了一个充满爱心、欢乐祥和的春节。这充分显示了中国特色社会主义制度的巨大优越性，体现了市委市政府以人为本、关注民生的执政理念，体现了全市上下万众一心、和衷共济、共赴时艰的强大凝聚力！

　　各位代表，回顾过去一年的工作，我们深感成绩来之不易。在此，我代表深圳市人民政府，向奋斗在各条战线的全市广大干部群众和外地来深建设者表示最崇高的敬意！向给予政府工作大力支持的人大代表、政协委员，向各民主党派、社会团体和各界人士，向中央和各省区市驻深单位，驻深解放军、武警官兵，向所有关心支持深圳建设的港澳同胞、台湾同胞、海外侨胞和国际友人，表示最衷心的感谢！

　　我们也清醒地看到，我市经济社会发展还存在一些亟待解决的困难和问题：一是产业结构调整优化任务十分艰巨，特别是要适应经济发展规律，在调整中科学把握好二三产业的良性互动和产业发展的消长平衡，难度很大。工业生产和固定资产投资增幅下降、物价持续上涨等问题也不容忽视；二是区域发展不平衡，特区外基础设施、环保设施、市政管理、公共服务欠账较多，城市化三年过渡期结束以后体制机制转型任务很重，促进特区内外一体化发展仍然需要付出巨大努力；三是一些群众关心的热点难点问题还没有得到有效解决，居民收入在社会分配中的比重偏低，资源和环境压力较大，维护社会和谐稳定的任务十分艰巨；四是安全生产形势不容乐观，基础工作不够牢固，各种事故隐患没有得到根本消除；五是政府部门的战略视野、工作标准、管理和服务水平离国际化城市的要求还有较大距离，社会管理和公共服务比较薄弱，民主法治建设有待加强，一些公务员的精神状态、工作作风、综合素质还有待进一步提高；六是人口总体素质不高，高端人才、技能型人才缺乏，等等。对这些问题，我们必须高度重视，认真采取措施，切实加以解决。

　　二、新的要求新的形势新的目标

　　2008年是深圳经济特区发展史上非同寻常的一年。这是全市上下按照党的十七大战略部署满怀信心阔步前进的开局之年，是深圳高扬经济特区旗帜，继续敢为人先、大胆探索、改革创新、争创一流的奋斗之年，是深圳以“特”的意识、“特”的思考、“特”的措施，率先探索完善中国特色社会主义制度模式，努力建设中国特色社会主义示范市的破冰之年，也是深圳以新一轮思想大解放推动新一轮大发展大跨越的启动之年。党中央国务院和省委省政府对深圳未来的发展寄以厚望，指明了方向，全市上下倍受鼓舞、整装待发！

　　面对中央的重托、全国的关注、世界的瞩目，政府深感责任重大、使命艰巨！唯有在市委的坚强领导下，在市人大、市政协和全市人民的监督、支持和帮助下，兢兢业业、恪尽职守，进不求名、退不避罪，求真务实、勇往直前，才能不负中央的信任，不负人民的期望！

　　当前，我市经济社会发展的外部环境趋于复杂，世界经济增长的不确定性因素明显增多。美国次贷危机的影响不断扩大，美元大幅贬值，爆发经济危机的可能性增大；全球经济面临衰退的风险，国际市场需求下降；原油、粮食、原材料等价格处于历史性高位，全球性通货膨胀正在蔓延；国际贸易保护主义抬头，贸易壁垒增多。深圳经济具有高度外向型特征，所受影响远大于其他城市，我们可能面临比1997年亚洲金融危机更加严峻的国际经济形势，一些不利影响已经在国际订单、企业成本和贸易摩擦中开始显现。

　　从国内环境看，国民经济运行中长期积累的一些突出矛盾和问题逐步显现，价格由结构性上涨演变为明显通货膨胀的压力进一步增大，金融市场风险加大。国家将进一步加强宏观调控，实施稳健的财政政策和从紧的货币政策，调整对外贸易政策。我市企业的基本面、资金面、政策面全面趋于紧张，经济面临国际、国内环境的双重挤压。同时，我们自身在经济社会运行和民主法治、民生保障等方面还存在许多内在矛盾和问题，对于前进中的困难，我们必须充分估计，勇敢面对，悉心把握，切不可掉以轻心。

　　尽管挑战空前严峻，但我们坚信，有党中央国务院和省委省政府的正确领导，有全体深圳人民的鼎力支持，有特区成立28年来积累的雄厚经济实力，有先行一步的体制机制优势，有改革开放创新的城市精神，只要我们万众一心、群策群力，解放思想、大胆探索，及早谋划、沉着应对，深圳一定能经受住新的考验，再创新的辉煌！

　　2008年政府工作的基本思路和主要任务是：以邓小平理论和“三个代表”重要思想为指导，以科学发展观统领经济社会发展全局，全面贯彻落实党的十七大、全国"两会"和省委十届二次全会精神，按照市委四届八次全会的总体部署，解放思想、改革创新、争创一流，用世界眼光和标准推动发展，加快推进改革开放和制度建设，加快推进产业升级和自主创新，加快推进深港合作和国际化城市建设步伐，加快提升城市品质和生态文明，加快改善民生和促进社会和谐，努力实现经济发展质量、效益与速度相统一，经济、政治、文化和社会建设“四位一体”协调发展，为争创中国特色社会主义示范市，探索中国特色社会主义制度模式做出新贡献。

　　综合考虑各方面的因素，2008年我市经济社会发展的主要预期目标和约束指标是：本市生产总值增长12%；城镇登记失业率控制在3%以内；居民消费价格总水平涨幅控制在4%左右；万元GDP建设用地下降10%，万元GDP能耗、水耗分别下降2.75%和4%；二氧化硫、化学需氧量排放量分别下降4%和3%。

　　需要说明三点：一是本市生产总值增长12%，主要是考虑了国际、国内多种因素对我市的影响，考虑了自身资源环境约束和产业优化升级的需要，考虑了保持经济平稳增长、促进经济与社会发展相协调的需要；二是万元GDP能耗、水耗分别下降2.75%和4%，主要是基于深圳当前万元GDP能耗、水耗已经处于较低水平，今年还将有一批重大工业项目相继投产，能耗、水耗继续大幅下降的空间不大，但是我们必须加强自我约束，通过认真实施节能减排，大力发展循环经济，使能耗、水耗继续保持下降趋势；三是居民消费价格总水平控制在4%左右，主要是考虑虽然通过加大调配储备力度，加强食品生产基地建设，实施财政补贴等措施，使去年我市的物价涨幅一直低于全国全省平均水平。但是我市市场化程度高，价格水平受外部因素影响较大，大部分资源、能源、原材料和农副产品供给来自市外，控制物价上涨难度很大；而考虑到经济社会正常运行的需要和居民、企业、社会各方面的承受能力，又必须努力把涨幅控制到一个相对合理的水平。

　　为实现上述目标，我们要通过深入开展新一轮思想大解放活动，寻求新一轮大发展的思路、对策和办法，要坚持稳中求进、又好又快的原则，坚持方向、力度、节奏相协调，做到速度、结构、质量和效益相统一，在工作中要把握宏观、积极调控，突出产业、增强后劲，完善基础、加强保障，关注民生、促进和谐，深化改革、搞活机制，突出重点、狠抓落实。

　　三、2008年主要工作

　　今年要着重抓好以下六个方面的工作：

　　（一）坚持高扬特区旗帜，加快推进改革开放和制度建设。

　　重塑经济特区改革开放新形象。以纪念改革开放30周年为契机，继续高扬经济特区的旗帜，认真总结改革开放的经验，弘扬敢闯敢试的精神，强化创新创业的激情，切实树立科学理念、忧患意识、全球眼光，敢于借鉴人类文明的共同成果，努力打破传统思维定势和行为定势，大胆从体制机制上进行改革攻坚，探索建立有利于科学发展、和谐发展，具有中国特色的经济社会管理体制和长效机制。改革要以民生为重，更多地问计于民，从人民群众中汲取改革的智慧和动力，更多地让普通群众了解改革、参与改革、支持改革、谋划改革，并实实在在地从改革中受益。要针对特区建设发展遇到的突出矛盾和问题，抓紧研究完善今年我市的改革计划，重点谋划和推动几项在全国有影响的重大改革措施，充分展示特区的崭新风采。

　　进一步理顺市场、社会和政府的关系。规范发展要素市场，培育完善市场机制；适应公民社会需要，深化社会领域的改革，培育社会自治能力；加强依法行政，规范政府行为，建立服务型政府。科学解决好政府与市场、社会之间存在的越位、错位和缺位问题，从体制上规范三者关系，逐步形成市场配置资源、社会自主管理、政府科学调控的善治格局，让市场发挥应有的作用，让社会更好地自主治理。在改革开放30周年之际重新审视和理顺三者的关系，具有更为重要的意义。

　　以深港合作为切入点全面推进体制创新。认真落实学习和追赶世界先进城市的决定，坚持以国际视野审视自己，以国际标准要求自己，清晰认识深圳所处的国际坐标，以全面推进深港合作为切入点，加快体制创新，提升国际化水平。深入贯彻“向香港学习，为香港服务，深港紧密合作，促进共同繁荣”的理念，全面学习借鉴香港融合西方文明与中华文化的制度设计，包括社会管理、公共服务、城市建设、政府运作、民主法治等，通过学习、消化、创新，形成具有中国特色、适应深圳市情的一系列制度创新，增创深圳体制机制新优势。全面加强“深港创新圈”建设，建立与香港的更紧密合作关系，促进双方在城市发展规划、重大跨境基础设施建设等领域的密切沟通与合作。更加主动、务实地推进深港合作项目建设，加快推进广深港客运专线、深港机场铁路接驳、莲塘/香园围口岸建设、皇岗和文锦渡口岸改造等重大项目的规划建设和前期工作，尽快启动深港河套地区的开发建设。适应经济发展需要，积极推动深港共建人民币离岸中心，促进两地资本市场融合，推动“深圳通”与香港“八达通”互联互通，促进人员往来便利化。继续加强与澳门的合作，落实深澳协商定期会议制度和政府高层定期会晤机制。

　　深化社会事业和社会管理体制改革。以提升公共服务水平为目标，在事业单位分类改革的基础上，加快推进法定机构试点，建立完善事业单位法人治理结构，改革事业单位人事制度和市属事业单位财政经费供给方式，实施事业单位岗位等级管理和绩效管理，建立公用事业专业化监管机构和公众监管委员会，改革公用事业产品价格调整程序与机制，建立健全公共事业管理体制和运行机制。加强社会工作者队伍建设，推进社会工作职业化和专业化，探索建立具有深圳特色的社会工作制度体系。引导、规范和鼓励社会力量举办公益事业，积极培育发展各类社会组织和中介组织，丰富公共产品和公共服务的供给。

　　深化行政管理体制改革。根据中央深化行政管理体制改革的精神，按照精简、统一、效能的原则，研究制定实施“大部门制”改革方案，大力推进政府机构及其职能的整合，不断完善政府组织体系和行政协调机制，探索以光明新区为试点，深化基层管理体制改革，形成权责一致、分工合理、决策科学、执行顺畅、监督有力的行政管理体制。深入推进行政许可和非行政许可审批制度改革，巩固改革成果，建立长效机制。深化政府投资项目建设管理体制改革，积极探索科学管理方式，提高建设效率、质量和管理水平。加快推进财税体制改革，加强公共预算管理，强化公共财政职能，逐步建立运作规范、透明高效的公共财政体制，促进公共财政更好地服务于经济发展方式的转变和产业结构的调整，更好地集中财力办大事，更好地改善民生和生态环境，让纳税人的钱更好地造福于社会和人民。

　　深化经济体制改革。进一步完善市场经济体系，营造公平竞争的市场环境。积极应对不利的国际经济形势，采取有效措施，降低市场交易和企业要素成本，支持帮助企业渡过难关。推动出台《深圳市促进中小企业发展条例》，支持和鼓励中小企业改制上市和发债融资，做好第二批中小企业集合债券的申报发行，探索成立产业投资基金，建立再担保机制和体系，改善中小企业融资条件。着力培育一批成长型中小企业，全力扶持民营领军骨干企业做强做大。继续深化国有企业内部各项改革，完善外部董事制度，健全企业法人治理结构，逐步建立与市场接轨的薪酬制度和劳动人事管理制度。学习新加坡“淡马锡”经验，探索将国资委设立为法定机构，完善出资人制度，创新国有资产运营和监管模式，增强国有企业竞争力和持续发展能力。积极开展资本运作，健全风险管理体系。积极推进集体经济和股份合作公司规范发展。

　　拓展对外开放的广度和深度。大力实施“走出去”战略。落实支持政策，完善政府服务，加强境外办展推介，发挥驻外机构和国际友城的作用，构建企业境外发展的全球经贸协作网络，协助优势企业积极开拓国内外市场，在保持外贸出口稳定增长的同时，提高企业国际竞争力，努力培育更多深圳本土跨国企业群体。稳步推进服务业对外开放，扩大服务出口，发展服务外包。大力推进越南中国（深圳）经贸合作区的建设和招商工作，打造全国境外合作区的样板项目。积极申报设立前海湾保税港区，推动盐田保税物流园区向综合保税区转型。研究利用CEPA条件探索自由贸易区试点工作。完善进出口贸易预警机制，指导、协助企业妥善处理各类国际贸易摩擦，维护产业和经济安全。加大重点项目招商工作，不断提高利用外资的质量和水平。

　　深圳的发展要面向全国，服务全国，在发挥特区示范辐射带动作用的同时，以服务创造深圳发展的新空间。这既是一种社会责任，也是深圳产业结构调整的内在需要。要加大对口扶持力度，加强与周边城市的合作，强化对欠发达地区的辐射和服务，认真办好异地深圳工业园和产业转移园。按照产业发展规律和市场规律，有序可控地引导部分产业扩张性转移。做好对外迁企业的调研分析和跟踪服务，加强对腾出土地的规划利用，真正实现“腾笼换鸟”，“长”大于“消”。积极创新区域合作模式，从管理体制、运行机制、统计与税收共享等方面加强制度创新与设计，以全新的思路探索与汕尾市的合作，拓展深圳发展空间，实现两地互利双赢。要进一步加强与珠三角和华南地区在基础设施、能源、经贸、旅游、劳务等方面的合作，积极参与西部大开发、振兴东北老工业基地、中部崛起和环渤海经济圈建设，实现共同发展。

　　（二）坚持好字优先，加快推进产业升级、自主创新和城市重大基础设施建设。

　　积极推动产业结构优化升级。要立足于深圳产业发展的基础和潜能，围绕提升国际竞争力，统筹规划推进产业转型升级，打造具有深圳特色的现代产业体系，形成高新技术产业、现代制造业与高端服务业良性互动、双轮驱动的格局。要突出高科技城市特色，抓核心技术、抓高端项目、抓品牌企业，推动高新技术产业向价值链高端延伸，提升深圳在国际产业分工中的地位。加快制定促进总部经济发展的具体政策，全方位优化总部企业发展环境，强力推进总部经济发展。加快中芯国际、深超光电、意法半导体封装测试、世纪晶源化合物半导体、赛诺菲巴斯德流感疫苗等项目建设步伐，以核心项目带动产业集群的发展。以国际一流标准推进高新区、光明高新园区、大工业区、精细化工园区、国家生物产业基地、航天科技产业基地和国家体育产业基地规划建设。大力推进汽车、光机电一体化等先进制造业发展，规划建设一批先进制造业总部基地。加强与军工科技企业合作，积极承接国家重大军工项目。鼓励优势传统产业加大技改投入，继续推进传统产业集聚基地建设，结合旧工业区改造，规划建设一批特色工业园区。培育和扶持远洋渔业发展，提升海洋产业发展水平。

　　加快建设国家创新型城市。积极推动创新促进条例的立法工作，大力完善区域创新体系，促进人才、技术、资金、管理等创新资源的聚集整合。改革科技管理体制，落实创新激励政策。加大科技研发资金投入特别是财政性资金投入，加快一批重点实验室等自主创新技术平台和公共服务平台建设。积极利用国内外科技资源壮大我市研究院群，增强基础应用研究和源头创新能力。制定完善“深港创新圈”建设战略规划，积极推进深港科技创新基地建设，促进深港两地创新资源整合与共享。进一步推动重点领域技术创新，采取项目带动的方式，通过政府资金引导、社会共同投入、跨学科跨部门联合攻关，力争实现若干关键技术的突破。积极发展化合物半导体、生物制药、智能机器人、下一代互联网等新兴战略创新产业，抢占未来产业发展制高点。积极推动医疗卫生、环境保护、交通运输、社会治安等社会民生领域的技术创新，提升民生福利水平。制定政府资助和采购自主创新产品的相关办法，加快发展创业投资，完善创新型企业成长路线图计划，着力培养一批具有强大自主创新能力的行业龙头企业。深入推进“人才强市”战略，进一步加大高层次人才培养引进力度，构建人才公共服务体系和人才市场体系，实施紧缺人才绿色通道，完善和落实人才政策，不断优化人才成长环境，切实提高我市对各类高端人才的吸引力。今年我市将再次组团赴欧洲和北美大规模招聘海外高端人才。加大对重大创新成果和创新人才的奖励，在全社会形成崇尚创新、自主创业的良好氛围。大力实施知识产权战略、品牌带动战略和标准战略，加大知识产权保护力度，继续推进国家知识产权示范城市建设。认真办好第十届“高交会”，充分发挥其创新资源交流平台的重要作用。

　　大力发展高端服务业。积极推动银行、证券、保险业创新发展，建设全国金融创新发展综合试验区。加快推进金融中心区和金融产业基地建设，进一步聚集金融资源。培育大型金融控股集团，积极推动深圳金融租赁公司战略重组，打造国内最大的金融租赁公司。适应金融业扩大开放的需要，加强与香港深层次金融合作，稳步推进与澳门的金融合作。完善多层次资本市场，为创业板的开设做好各项准备，积极争取在高新区开设股份代办转让系统，进一步推进华南技术产权交易市场建设。加快培育发展企业债券市场，进一步巩固我市基金业在全国的龙头地位。积极维护金融市场秩序，切实防范和化解金融风险。

　　提升现代物流业发展水平。大力加强物流基础设施建设，完善物流产业链条。发展多式联运，继续推进海铁联运和江海联运，实施“珠江战略”，拓展深圳港经济腹地。吸引国际班轮公司地区性总部或操作中心落户深圳，不断增强深圳港作为国际集装箱枢纽港的辐射力和竞争力。积极推动深圳机场与香港、广州机场的战略合作，鼓励、支持现有基地航空公司增开国际航线，加快推进空港保税物流园区申报。建立完善陆路物流运输体系，促进陆路物流规模化、集约化、现代化、品牌化，推动陆海空港同步协调发展。积极促进物流资源整合，做大做强一批物流企业。

　　培育发展现代商业新型业态，加快构建覆盖面广、结构合理、活跃繁荣、辐射力强的城市商圈体系。不断改善消费环境，拓展消费领域，提升居民消费层次。加快实施高端服务业企业认定办法和资助计划，继续壮大旅游、会展、信息服务业，积极发展会计、律师、咨询、设计、创意等专业服务业。

　　加大固定资产投资力度。集中财力、确保重点、科学安排、统筹兼顾，着力推进事关未来发展后劲的重大产业项目、事关城市竞争力和辐射力的重大基础设施、事关民生福利、事关特区内外一体化建设、事关经济社会发展安全的重大项目的建设。加快推进大铲湾、盐田港区深水泊位和西部港区公用航道建设，完善港口集疏运体系和物流支持体系。加快推进深圳机场飞行区和航站区扩建及配套工程。全力推进轨道交通二期工程5条线路及相关交通综合枢纽建设。积极推动国家铁路“两线两站”规划建设，加快设立广深港铁路光明新区客运站。抓紧高快速路、城市主干道路网建设和特区外重点地区的交通综合治理及微循环改造，建成东部沿海高速公路莲塘至盐田段、盐坝高速公路C段，积极推进南坪快速路二期、丹平快速路一期、布龙公路城市化改造、沿江高速公路深圳段等工程。

　　（三）坚持以人为本，加快推进以改善民生为重点的社会建设。

　　加大民生领域的政府投入。发展依靠人民，发展为了人民。作为国内人均GDP最高的城市，深圳市民有权利实实在在地分享到发展的成果，深圳人的民生福利水平应该是全国最高的，政府必须为此不懈努力！要继续办好顺应民意、化解民忧、为民谋利的实事好事，努力提供更多更好的公共产品和公共服务，显著提升民生福利水平，让发展成果惠及全体市民。2008年，全市财政一般预算支出的3/4将安排在公共服务和民生领域，总投入554.9亿元，所占比重比去年提高2.7个百分点。政府投资计划中民生领域的投资223亿元，占74.4%，比重进一步增加。

　　努力提高居民收入水平。处理好效率和公平的关系，强化税收等调节手段，逐步建立更加合理的收入分配制度，提高劳动报酬在初次分配中的比重。实行积极的工资政策，加强企业工资宏观调控，建立企业职工工资正常增长机制，广泛推行工资集体协商制度，着力提高最低工资和低收入劳动者工资水平。严厉打击恶意欠薪和逃薪行为。高度重视物价问题，加大市场监管力度，坚决打击哄抬物价等违法行为，严格控制关键性商品和服务的价格调整计划，确保市场价格总水平运行平稳。建立困难群体生活补贴与物价水平联动机制，确保困难群众的生活水平不因物价上涨而降低。

　　积极打造充分就业城市。积极贯彻实施《劳动就业促进法》，进一步强化就业再就业工作，大力实施积极的就业政策，鼓励自主创业，力争年内80%的社区成为充分就业社区。加强劳动者就业培训，鼓励和引导本地居民就业，完善面向困难群体的就业援助制度，确保"零就业"家庭出现一户解决一户。积极稳妥地贯彻实施《劳动合同法》和《劳动争议调解仲裁法》，及时反映和解决实施中出现的问题，加强和健全区、街道两级劳动仲裁力量，引导企业加强制度建设，依法保护劳资双方合法权益，积极构建和谐劳动关系。

　　继续推进教育均衡发展。认真贯彻国家免费义务教育政策，结合深圳实际尽快出台实施办法。落实义务教育均衡发展督导方案，积极推动特区内外教育管理体制一体化。加快特区外96所原村办小学改造进程，确保明年全部完成改造。高度关注中小学生健康成长，全面落实素质教育，年内妥善解决好学生在校午餐午休问题。召开学前教育工作会议，完善相关政策，推动学前教育健康发展。加快寄宿制高中学校建设，大力发展职业教育，加强高素质技能人才培养。努力提升大学教育发展水平，适当扩大市属高校生源范围。强力推进南方科技大学各项建设和筹办工作，争取早日具备办学条件，早日建成招生。提升深圳大学等高等院校教学科研水平，加快深圳大学扩建和医学院建设步伐，支持深圳职业技术学院和深圳信息职业技术学院建设国家示范性学院。认真总结完善大学城办学体制，更好地发挥大学城在知识创新和科学研究中的重要作用。规范民办教育发展，加大民办学校资源整合。积极推进国际学校建设，加强国际教育交流。构建终身教育体系，建设学习型城市。

　　完善均衡化公共医疗卫生服务。强化公共医疗卫生事业的公益性质，加大公共医疗卫生事业的投入。继续实施“一大一小”战略，今明两年将动工兴建4个市级医院，做到每个区有一个市级医院，让市民就近享受到高水平的医疗服务。继续推进滨海医院、疾控中心等公共卫生重点项目建设，加快基层医院改造进度。建立完善社区健康服务网络和机制，实施依托大医院的院办院管体制，提高医疗卫生服务水平。继续深化医疗卫生体制改革，鼓励民营医院加快发展。加快建立基本医疗卫生制度，改进医德医风。进一步打造人口和计划生育公共服务体系，稳定低生育水平，提高出生人口素质。积极开展爱国卫生运动，加快发展妇幼卫生事业。

　　同心协力加快破解交通拥堵这一世界性难题。全力以赴加快轨道交通建设进度，确保到2011年前形成总长度177公里的轨道交通网络，实现特区内30分钟内、特区外其它城市组团45分钟内到达市中心区的目标，大大改善市民的交通出行环境。今年是轨道交通建设的关键期和高峰期，全市将有109个地铁站、104个建设区间、约150个施工点同时开工，深圳交通将因此经历一年半最困难的时期。为了减轻轨道交通建设给市民带来的出行影响，政府将进一步提高交通科学管理水平，加大交通疏解力度，采取多种措施将施工带来的交通拥堵降到最低点。同时，恳请市民群众更多地使用公共交通工具，注意政府发布的交通信息，尽量避开拥堵路段、错开高峰时段出行，文明礼让，遵章驾驶，并对政府工作给予理解和支持。继续实施公交优先战略，加快公交特许经营改革，完成特区外公交整合工作，加强行业监管，提高公交服务水平。进一步完善公交补贴机制，政府增加补贴2000万元左右，年内实现全市范围内65岁以上老年人免费搭乘公交车。加大公交场站建设力度，优化特区外公交线网布局，新开一批公交线路，特区外建成区公交500米覆盖率要达到70%以上。严厉整治非法营运行为，维护良好交通营运秩序。

　　进一步完善社会保障体系。认真实施《社会保险法》，深化养老、失业和医疗保险制度改革，不断完善全民社保制度。适度提升社会保险待遇，稳步提高保障水平，让参保人享受更多实惠。继续扩大社会保险覆盖面，确保工伤和医疗保险参保人数达到760万以上，少儿医疗保险参保人数达到40万以上。每年拿出2000万元补贴残疾人社保，年内实现全市残疾人社会保险全覆盖。同时研究将家政服务业、建筑行业从业人员以及更多外来劳务工纳入保障体系。积极配合国家完善社保关系转移接续制度，妥善解决好劳务工退保问题。加快建立完善以最低生活保障制度为核心的跨部门综合性社会救助体系，建立健全社会帮扶体系。大力发展社会福利、慈善捐赠、老年人和残疾人事业，推进慈善组织和工作网络建设，实现残疾人“人人享有康复服务”。实施第四期同富裕工程，进一步促进欠发达地区的发展。完善住房保障政策，重点发展普通商品住房和各类保障性住房，更好地解决中低收入者住房需求问题。优先确保各类保障性住房用地，加快梅山苑二期、松坪村三期及地铁上盖等保障性住房项目建设，争取保障性住房供应总量达到年度住房供应总量的20%以上，确保廉租住房保障对“双困家庭”的全覆盖。继续加强房地产市场秩序整治，促进房地产市场平稳健康发展。

　　大力维护社会和谐稳定。安全利益是老百姓的第一利益，要认真吸取“2·27”火灾事故教训，穷尽排查、铁腕整治、固化责任、强化机制，完善安全生产责任体系与综合监管体系建设，重点整治和长效管理相结合，积极开展安全隐患排查整治活动，全面推行中小企业安全生产托管制度，切实强化安全生产企业法人责任和政府监管责任，大力普及安全知识，坚决防止重特大安全事故的发生。强化社会治安综合治理，落实综合治理责任制，严厉打击各类刑事犯罪，努力实现社会治安持续好转。深入推进公安专业化改革，加强科学化、规范化管理，强化科技强警工作，不断提高公安队伍正规化建设水平。加强人口管理与服务，全面推行居住证制度，加大居住证宣传力度，弱化户籍概念，强化居民意识，使持证人在工作、生活、子女教育等方面更好地享受到公共服务便利。全力推进产品质量和食品药品安全工作，加快食品安全“五大工程”、“四大体系”建设，大力整顿和规范食品、药品市场秩序，不断提高老百姓的饮食、用药安全水平。着力推进菜篮子工程，进一步提高全市农产品保障能力。探索建立日用化学品安全评价机制。建立完善市场监管制度，积极推进社会诚信体系建设。加强维稳和信访工作，完善市政府12345公开电话系统，进一步健全社会利益协调机制、诉求表达机制和矛盾调处机制。

　　进一步提升城市应急能力和水平。大力加强应急管理体制机制建设，提高预测预警和应急救援能力，积极预防和妥善处置各类突发公共事件。完善卫生应急机制，提高公共卫生事件应对能力和重大疾病预防控制能力。加强联动联防，加快城市防灾减灾工程建设，提高应对极端气候变化、自然灾害和防灾减灾、应急保障能力。

　　积极发展其他各项社会事业。进一步改革完善社区管理体制，明确社区工作站定位，做到权、责、利相统一，不断提升基层管理和自治水平。认真组织好2008年第五届居委会换届选举工作，确保实现直选率70%的目标。积极推进物业管理与服务的规范化建设。完善优抚安置保障体系，巩固和加强军政军民团结。完善档案法规和规章制度，加快推进档案信息化、数字化，建设现代化的一流档案馆和档案集中储存基地。进一步加强民族宗教、外事、港澳台务、侨务和民防工作。

　　（四）坚持文化立市战略，加快推进和谐文化建设。

　　努力提高城市文明水平。认真落实中央关于推动社会主义文化大发展大繁荣的部署，把社会主义核心价值体系建设作为文化建设的中心环节来抓，积极探索中国特色社会主义文明模式。加快文化创新，不断提升深圳的文化软实力。繁荣新闻出版、广播影视和文学艺术事业，大力发展哲学社会科学，加强对深层次、基础性、前瞻性问题的研究。以增强诚信意识和责任意识为重点，切实加强社会公德、职业道德、家庭美德、个人品德建设，加大公益广告宣传力度，提高市民文明素质，提升城市文明程度，迎接第二届全国文明城市评选。深入开展“关爱行动”等各种形式的和谐创建活动，完善社会志愿者服务体系，加强人文关怀和心理疏导，塑造自尊自信、互爱互助、理性平和、积极向上的公民社会心态。着力推动基层和谐文化建设，以和谐社区、和谐企业、和谐家庭建设为载体，不断增强市民城市认同感和归属感，培育家园意识。加大普法宣传力度，提高市民的法制意识。

　　积极推动文化事业发展。进一步完善公共文化设施网络，加快构建覆盖全社会的公共文化服务体系，切实提高公共文化服务质量。围绕纪念改革开放30周年，积极推动创作一批文艺精品。争取年内建成博物馆新馆，加快当代艺术馆、城市规划展馆的建设，做好歌剧院前期论证，加大特区外社区文体设施建设力度。进一步办好各类品牌文化活动，大力开展周末系列、流动系列和高雅艺术系列文化活动，提高市民的公共文化参与度，不断增进市民群众和广大劳务工的文化福利。加大对咸头岭遗址、大鹏所城、南头古城、客家民居等文物古迹和传统建筑的保护力度，加强对传统民俗风情等非物质文化遗产的开发和保护，进一步增强城市发展的历史感和厚重感。积极促进深港文化交流，广泛开展对外文化交流活动，加强城市国际形象与国际品牌建设，提高城市的国际知名度。

　　大力发展文化产业。全面实施文化产业发展规划纲要，落实文化产业配套政策，加快推进“两城一都一基地”建设，促进文化产业跨越式发展，努力建设文化产业发展的中心城市和先锋城市。加强特色文化产业园区和基地建设，鼓励旧工业区、旧村和旧城区改造建设成文化产业园区。巩固发展广告会展、现代印刷等优势传统文化产业，重点扶持创意设计、动漫游戏、数字视听、新媒体等与高新技术产业相融合的新兴文化产业，打造一批具有核心竞争力的文化产品和文化品牌，提高文化产业在全市经济结构中的比重。引入香港品牌机构参与举办第四届“文博会”，提高“文博会”的国际化、专业化、市场化水平。积极发展高端旅游，全面启动深圳湾15公里海滨休闲带和金沙湾建设，加快东部华侨城二期、欢乐海岸项目建设，积极推进太子湾国际邮轮母港和未来乐园的建设。

　　全力做好第26届世界大学生运动会各项筹备工作。科学规划、高效率、高质量推进大运中心、大运村、南山体育中心、宝安体育场、航海运动学校等大运会场馆及相关项目建设，努力打造环境一流的大运新城。办好“大运文化节”，充分调动全体市民和社会各界的参与热情，为迎接大运会营造良好的社会氛围。组织好2008北京奥运火炬传递工作，大力开展群众性体育活动，积极承办国际高端赛事。

　　（五）坚持可持续发展，加快推进城市品位建设和生态文明建设。

　　着力优化城市功能布局。加强城市发展战略和区域协调发展研究，以世界眼光和国际一流水准，全面启动“十一五”规划中期评估和修编工作，尽快完成城市总体规划和土地利用总体规划编制，加快实现法定图则全覆盖。进一步完善城市规划体系和城市规划实施机制，加强各层次、各领域规划的衔接和协调，发挥城市规划对城市建设和土地利用的引导、统筹和主导作用。加强重点地段和重点区域城市设计，开展城市美化行动，集中规划建设一批城市景观和建筑精品，重点推进生态文明建设系列工程，不断提升城市形象和气质。充分发挥政府主导作用，动员社会力量参与，加大城市更新力度，统一规划，完善政策，强力推进城中村、旧工业区、旧商业区和旧居住区改造升级，加快闲置土地清理和各类土地遗留问题处理，盘活存量用地资源。创新产业用地管理，根据产业周期合理确定土地出让年限，加快土地周转，提高利用效率。强化市政府对土地的集中统一管理，尽快完成宝安、龙岗两区和光明新区城市化转地收尾和后续管理工作，严格土地储备管理。严控建设用地增量，切实落实和保护基本农田。推进城市地下、空中和海洋空间资源的调查研究、规划管理和审慎开发。

　　深入推进特区内外一体化发展。今年起，宝安、龙岗和光明新区的道路、环保等市政设施全面移交区级政府维护和管理。进一步加大特区外基础设施投入和建设力度，积极改善特区外生产生活条件，切实做好特区外城市化过渡期后的体制转轨工作，提高特区外市政管理和公共服务水平。以光明新区中心区建设为突破口，按照达到甚至高于特区内现有水平的标准，深度规划，重点投入，加强管理，加快推进四大新城的开发建设，力争把四大新城和宝安、龙岗中心区建成缩小特区内外差距，实现均衡发展的示范区，并带动其他区域全面发展。六区和光明新区要努力避免功能和产业的同质化，充分发挥各自特色，找准定位，实现差异化发展。

　　大力加强生态建设与环境保护。牢固树立生态政绩观，强力推进生态市建设，积极开展"绿色政府"创建活动，健全环境与发展综合决策制度，实施领导干部环保实绩考核办法，切实加强环保责任约束，努力打造强势环保。加快污染减排的指标、监测和考核三大体系建设，完善治污保洁工作机制，进一步加大环保监测和执法力度。深入开展深圳河、龙岗河、观澜河、茅洲河、大沙河等重点流域的综合整治。未来几年将投入106亿元，加快推进27座污水处理厂和580公里污水截排收集主管网、600公里支管网系统建设，同时继续深入开展排水管网清源活动，尽最大努力两年内实现建成区污水的全部收集和处理。继续实施蓝天工程，推进大气污染、噪声污染治理，提升固体废弃物处理能力。建立完善生态补偿机制，加大基本生态控制线监测和保护力度，加强水土保持和采石取土场所整治复绿，深入推进生态风景林和水源保护林建设，开展节约型园林绿化，构建生态安全格局。在光明新区开展绿色城市规划和绿色建筑试点，积极推进国家生态区、生态工业示范园区、生态街道等系列创建活动。严格执行建筑节能标准，积极开展绿色建筑认证和建筑节能改造，全面推行绿色设计和绿色施工，打造绿色建筑之都。

　　努力建设资源节约型、环境友好型社会。积极发展循环经济，强力推进节能减排，创新环境经济政策，加强节能减排执法，建立推广产业能耗指导标准，实施重大项目节能评估和审查制度，综合运用市场、价格、信贷、环保和政府资助等手段，引导企业和重点行业实行清洁生产、节能降耗和资源综合利用。大力发展节能环保产业，促进建筑垃圾、生活垃圾和工业固体废弃物的综合利用。积极开发节约、替代、循环利用和污染治理的先进适用技术，发展太阳能、风能等清洁能源和可再生能源，推进生物化工技术研究应用。大力推广循环经济试点示范项目，推进住宅产业化综合试点城市建设，加快形成节约能源资源和保护生态环境的产业结构、增长方式和消费模式。

　　按照“科学、严格、精细、长效”的要求，全面提升城市管理水平。进一步完善街道综合执法体制，逐步理顺各方关系，合理划分综合执法事项，完善综合执法环境。丰富和创新城市管控手段，完善数字化城管系统，加大网格化管理覆盖面，全面提升城市管理的精细化和信息化水平。加大城市公共设施建设力度，加快建设垃圾中转站及无害化处理设施、大型环卫设施，加强城市建筑和市政设施的后续管理和综合整治，深入推进城中村和社区环境综合治理。高标准建设红树林湿地生态修复工程，继续推进各类公园和自然保护区建设，创新公共设施和公园管理体制，切实满足市民的休闲需要。继续落实查违工作责任制，强力查处违法建筑，确保实现违法建筑“零增量”。加大力度处理历史遗留违法建筑问题，强化“清无”行动的后续管理。

　　进一步完善资源能源保障体系。继续推进东江水源工程二期等水源网络和公明、铁岗、清林径、东江下矶角等水务工程建设，将全市原水年供应能力提高到19亿立方米。加强城市供水设施建设，改造陈旧市政供水管网，尽快废除镀锌管。实施城市水系蓝线管理，积极推进水生态修复和水景观建设，对一级水源保护区实行封闭管理。强化计划用水和建设项目用水节水管理，开展雨洪、地下水、海水和中水资源的科学利用，建设海水淡化示范工程，实施再生水源开发和战略储备，建立循环利用的水资源保障体系，加快推进节水型城市的创建。进一步改革完善水源管理体制，整合特区内外水资源管理。加快推进岭澳核电三期工程和滨海电厂项目前期工作，积极落实电网建设规划，抓紧完成抽水蓄能电站、骨干环网等电源、电网工程建设。积极争取省网供电指标和区外电力供应，继续实施燃油燃气发电补贴措施，调动地方机组顶峰发电。强化电力需求侧管理，完善大面积停电处置预案和"黑启动"机制。积极推进垃圾焚烧发电、风力发电、太阳能光伏系统等新兴能源项目的研究论证和规划建设。推进建立多层次的成品油、气储备体系，加快深圳第二个LNG接收站、国家成品油深圳储备基地、天然气安全储备库等项目的规划建设，积极推进西气东输工程和城市天然气输配管网建设，全面完成居民用户天然气转换。全面落实与成品油供应企业的战略合作，进一步完善成品油供应网络和市场应急预警机制。加强粮食等重要物资的储备和调控管理。

　　（六）坚持依法行政，加快推进服务型政府建设。

　　进一步转变政府职能。健全政府职责体系，全面正确履行政府职能，减少和规范行政审批，强化和完善社会管理和公共服务，更好地发挥市场和社会的作用。按照行政责任体系“1＋6”文件要求，完善行政内部监督机制，强化对政府部门履职情况、政府重大政策落实情况和重大项目进展情况的监督监察。继续强化政府目标管理，推广部门责任“白皮书”制度，完善政府部门绩效评估，进一步扩大绩效审计范围，对政府各部门履行法定职责、完成工作目标、使用财政资金、依法行政以及工作效率等方面的情况进行综合考评。大力推行行政首长问责制，切实加强行政问责。进一步完善行政审批体系，强化联动审批机制，加快在线行政审批系统建设，完善电子监察系统，努力提高政府效能。进一步提高行政服务大厅服务水平，打造一流的综合行政服务机构。加强政务信息化建设，强化部门间系统互动和信息资源共享，加快信息安全保障体系建设，不断提高政府行政效率和信息化水平。

　　大力加强民主法制建设。认真接受市人大及其常委会的法律监督和工作监督，坚持定期向市人大常委会通报重要工作。认真接受政协委员的民主监督，加强与市政协的工作沟通和协商，主动听取各民主党派、人民团体和社会各界的意见和建议。切实执行人大通过的各项决议，认真办理人大建议和政协提案、建议案。学习借鉴国际先进法治经验，加强政府立法工作，提高依法行政水平。推出法治政府建设考评指标体系，全面推进法治政府建设，力争3年内率先实现国务院提出的建设法治政府的各项目标。健全科学民主的决策机制，积极推进实施政务公开和"阳光工程"，最大限度地保障人民群众的知情权、参与权、表达权和监督权。切实提高政府部门的公共关系意识和公共关系工作水平，在制定和实施与群众利益密切相关的规章和公共政策时，一定要了解民情、倾听民意，制订公共关系工作预案，不断完善公众有序参与机制，促进政府与市民的沟通和良性互动。

　　加强公务员队伍管理和政风建设。探索公务员职位分类管理，深入推进聘任制公务员改革试点，逐步建立科学、规范、更有活力的公务员管理体制。以提升能力素质、法制素质、民本素质为重点，加强公务员学习培训，建设一支政治坚定、业务精湛、作风过硬、人民满意的公务员队伍。强化考核监督机制，健全完善公务员权益保障和激励机制，规范公务员津贴补贴。统筹协调对基层的考核检查和评比活动，尽可能减轻基层负担。切实加强调查研究，树立求真务实的工作作风。保持艰苦奋斗、勤俭节约的优良传统，努力降低行政成本。

　　扎实推进廉政建设工作。坚持标本兼治、综合治理、惩防并举、注重预防的方针，以完善惩治和预防腐败体系为重点，深化改革，创新体制，拓展从源头上防治腐败的工作领域，强化权力制约和监督，努力形成拒腐防变教育长效机制、反腐倡廉制度体系和权力运行监控机制，切实提高反腐倡廉建设成效。深入推进治理商业贿赂专项工作，坚决纠正损害群众利益的不正之风，依法严惩腐败分子，树立清正廉明、干净干事的政府形象。

　　各位代表！

　　深圳经济特区是改革开放的一面旗帜，是展示中国特色社会主义风采和中国美好未来的一个窗口。责任重大，使命光荣！让我们高举中国特色社会主义伟大旗帜，更加紧密团结在以胡锦涛同志为总书记的党中央周围，以科学发展观统领全局，在市委的坚强领导下，团结全市人民，进一步解放思想，放眼世界，开拓创新，奋勇争先，为建设中国特色社会主义示范市，为建设我们更加美好的共同家园而努力奋斗！
